# Supplementary material for: Senotherapeutic drugs for human intervertebral disc degeneration and low back pain
Source: eLife. 2020 Aug 21;9:e54693. doi: 10.7554/eLife.54693 (PMC7442487; doi:10.7554/eLife.54693)
Supplement: Supplementary file 2. [file elife-54693-supp2.docx]

**Supplementary File 2. List of cytokines and synonyms.**

| **#** | **Name** | **Symbol** | **Synonyms** |
| --- | --- | --- | --- |
| **1** | Angiogenin | Ang | Ribonuclease 5 |
| **2** | Brain-derived neurotrophic factor | BDNF |  |
| **3** | B lymphocyte chemoattractant | BLC | CXCL13 or BCA-1 |
| **4** | Chemokine (C-C motif) ligand 23 | CCL23 | CKβ8 / CKβ8-1/ MIP-3 / MPIF-1 / SCYA23 |
| **5** | Epidermal growth factor | EGF |  |
| **6** | Epithelial neutrophil- activating protein 78 | ENA-78 | CXCL5 |
| **7** | CC chemokine subfamily 1 of eosinophil chemotactic proteins. | Eotaxin-1 | CCL11 |
| **8** | CC chemokine subfamily 2 of eosinophil chemotactic proteins. | Eotaxin-2 | MPIF-2 / CCL24 |
| **9** | CC chemokine subfamily 3 of eosinophil chemotactic proteins. | Eotaxin-3 | CCL26 |
| **10** | Fibroblast growth factor 4 | FGF-4 |  |
| **11** | Fibroblast growth factor 6 | FGF-6 |  |
| **12** | Fibroblast growth factor 7 | FGF-7 | KGF |
| **13** | Fibroblast growth factor 9 | FGF-9 |  |
| **14** | Fms-like tyrosine kinase 3 | Flt-3 Ligand |  |
| **15** | Chemokine [C-X3-C motif] ligand 1 | CX3CL1 | Fractalkine |
| **16** | Granulocyte chemotactic protein 2 | GCP-2 | CXCL6 |
| **17** | Granulocyte colony stimulating factor | GCSF | G-CSF / CSF 3 |
| **18** | Glial cell-derived neurotrophic factor | GDNF |  |
| **19** | Granulocyte-macrophage colony-stimulating factor | GM-CSF |  |
| **20** | Chemokine (C-X-C motif) ligand 1 | CXCL1 | GRO-α, KC, NAP-3, MGSA-α |
| **21** | Growth-regulated oncogene- a/b/g | GRO α/β/γ |  |
| **22** | Hepatocyte growth factor | HGF |  |
| **23** | Chemokine (C-C motif) ligand 1 | CCL1 | TCA-3 / I-309 |
| **24** | Interferon gamma | IFN-γ |  |
| **25** | Insulin growth factor 1 | IGF-1 |  |
| **26** | Insulin growth factor binding protein 1 | IGFBP-1 |  |
| **27** | Insulin growth factor binding protein 2 | IGFBP-2 |  |
| **28** | Insulin growth factor binding protein 3 | IGFBP-3 |  |
| **29** | Insulin growth factor binding protein 4 | IGFBP-4 |  |
| **30** | Interleukin 1α | IL-1α | IL-1 F1 |
| **31** | Interleukin 1β | IL-1β | IL-1 F2 |
| **32** | Interleukin 2 | IL-2 |  |
| **33** | Interleukin 3 | IL-3 |  |
| **34** | Interleukin 4 | IL-4 |  |
| **35** | Interleukin 5 | IL-5 |  |
| **36** | Interleukin 6 | IL-6 |  |
| **37** | Interleukin 7 | IL-7 |  |
| **38** | Interleukin 8 | IL-8 | CXCL8 |
| **39** | Interleukin 10 | IL-10 |  |
| **40** | Interleukin 12 | IL-12 | p40/p70 |
| **41** | Interleukin 13 | IL-13 |  |
| **42** | Interleukin 15 | IL-15 |  |
| **43** | Interleukin 16 | IL-16 |  |
| **44** | Interferon gamma-induced protein 10 | IP-10 | CXCL10 |
| **45** | Leptin: Hormone promotes inflammatory responses | Leptin |  |
| **46** | Leukemia inhibitory factor (IL6 class cytokine) | LIF |  |
| **47** | Tumor necrosis factor superfamily member 14 | TNFSF14 | LIGHT |
| **48** | Monocyte chemoattractant protein 1 | MCP-1 | CCL2 |
| **49** | Monocyte chemoattractant protein 2 | MCP-2 | CCL8 |
| **50** | Monocyte chemoattractant protein 3 | MCP-3 | MARC / CCL7 |
| **51** | Monocyte chemoattractant protein 4 | MCP-4 | CCL13 |
| **52** | Macrophage colony-stimulating factor | M-CSF |  |
| **53** | Macrophage-derived chemokine, a CC chemokine | MDC | CXCL9 |
| **54** | Macrophage migration inhibitory factor | MIF | MMIF |
| **55** | Monokine induced by gamma interferon | MIG | CXCL9 |
| **56** | Macrophage Inflammatory Protein 1β | MIP-1β | CCL4 |
| **57** | Macrophage Inflammatory Protein 1δ | MIP-1δ | CCL15 |
| **58** | Macrophage inflammatory protein 3 α | MIP-3α | CCL20 |
| **59** | neutrophil-activating peptide | NAP-2 | PPBP / CXCL7 |
| **60** | Neurotrophin-3 | NT-3 |  |
| **61** | Neurotrophin-4 | NT-4 |  |
| **62** | Oncostatin M | OSM |  |
| **63** | Osteopontin | OPN | SPP1/ BSP-1/ BNSP |
| **64** | Osteoprotegerin | OPG | TNFRSF11B/ OCIF |
| **65** | Pulmonary and activation-regulated chemokine | PARC | CCL18/ DC-CK1/ MIP-4 |
| **66** | Platelet-derived growth factor-BB | PDGF-BB |  |
| **67** | Placental Growth Factor | PLGF |  |
| **68** | Regulated upon Activation, Normal T cell Expressed, and Secreted | RANTES | CCL5 |
| **69** | Stem cell factor | SCF | KIT-ligand/ KL/ steel factor |
| **70** | stromal cell-derived factor | SDF-1α | CXCL 12α |
| **71** | Thymus- and activation-regulated chemokine | TARC | CCL17 |
| **72** | Transforming growth factor beta 1 | TGF-β1 |  |
| **73** | Transforming growth factor beta 2 | TGF-β2 |  |
| **74** | Transforming growth factor beta 3 | TGF-β3 |  |
| **75** | Thrombopoietin | THPO | TPO/ MGDF |
| **76** | Tissue inhibitor of metalloproteinases 1 | TIMP-1 |  |
| **77** | Tissue inhibitor of metalloproteinases 2 | TIMP-2 |  |
| **78** | Tumor necrosis factor-α | TNF-α |  |
| **79** | Tumor necrosis factor-β | TNF-β | TNFSF1B |
| **80** | Vascular endothelial growth factor-α | VEGF−α |  |
