# Supplementary material for: Senotherapeutic drugs for human intervertebral disc degeneration and low back pain
Source: eLife. 2020 Aug 21;9:e54693. doi: 10.7554/eLife.54693 (PMC7442487; doi:10.7554/eLife.54693)
Supplement: Supplementary file 3. — (a) Eight Differentially Expressed genes significantly up- or downregulated at p < 0.05 in RG-7112 condition. (b) Forty Differentially Expressed genes significantly up- or downregulated at p < 0.05 in o-Vanillin condition. (c) Non-Differentially Expressed genes in RG-7112. (d) Non-Differentially Expressed genes in o-Vanillin. [file elife-54693-supp3.docx]

**Supplementary File 3a.** Eight Differentially Expressed (DE) genes significantly up- or downregulated at p < 0.05 in RG-7112 condition.

| **Decrease** |
| --- |
| **Increase** |

| **Target Name** | **Fold change Average in RG-7112 / CTRL** | **p value** |
| --- | --- | --- |
| MDM2-Hs01066930_m1 | 0.087305103 | 9.30709E-06 |
| CDKN1A-Hs00355782_m1 | 0.109616102 | 0.000463295 |
| MAPK14-Hs00176247_m1 | 1.920704132 | 0.00432843 |
| CCNB1-Hs01030099_m1 | 3.029621193 | 0.0164353 |
| CDKN2D-Hs00176481_m1 | 8.941050386 | 0.00254532 |
| E2F1-Hs00153451_m1 | 11.8956226 | 0.0252918 |
| RBL1-Hs00765700_m1 | 13.43390671 | 0.0106087 |
| CDC25c-Hs00156411_m1 | 123.2215587 | 0.0417112 |

**Supplementary File 3b.** Forty Differentially Expressed (DE) genes significantly up- or downregulated at p < 0.05 in o-Vanillin condition.

| **Decrease** |
| --- |
| **Increase** |

| **Target Name (DE Genes)** | **Fold change Average in O-Vanillin / CTRL** | **p value** |
| --- | --- | --- |
| VIM-Hs00185584_m1 | 0.007227241 | 0.00496146 |
| FN1-Hs00365052_m1 | 0.01259738 | 0.000387542 |
| CD44-Hs01075861_m1 | 0.041070857 | 0.0137907 |
| THBS1-Hs00962908_m1 | 0.186501397 | 0.0401026 |
| SOD2-Hs00167309_m1 | 0.308665425 | 7.90902E-05 |
| CCNB1-Hs01030099_m1 | 0.665558586 | 0.0184637 |
| PLAU-Hs01547054_m1 | 0.674640648 | 0.0149963 |
| CCNA2-Hs00996788_m1 | 0.780636981 | 0.0121212 |
| CDKN2A-Hs00923894_m1 | 0.796208568 | 0.00294665 |
| CCND1-Hs00765553_m1 | 0.942854208 | 0.00172722 |
| BCL2L1-Hs00236329_m1 | 1.004240088 | 0.00544705 |
| TGFB1-Hs00998133_m1 | 1.206506403 | 0.000190618 |
| CDK6-Hs01026371_m1 | 1.218291962 | 0.00857665 |
| CHEK1-Hs00967506_m1 | 1.227071381 | 0.0189981 |
| ETS1-Hs00428293_m1 | 1.265775467 | 0.00449578 |
| AKT1-Hs00178289_m1 | 1.346969195 | 0.00663283 |
| IRF3-Hs01547283_m1 | 1.478236795 | 0.00590973 |
| HRAS-Hs00978050_g1 | 1.559048713 | 0.0131928 |
| MAPK14-Hs00176247_m1 | 1.796801852 | 0.0204895 |
| MAP2K3-Hs00177127_m1 | 2.619252609 | 5.40303E-05 |
| NFKB1-Hs00765730_m1 | 2.917191868 | 0.0013348 |
| CDKN2C-Hs00176227_m1 | 2.927476113 | 0.00193099 |
| ABL1-Hs01104728_m1 | 2.972531628 | 1.98834E-05 |
| TGFB1I1-Hs00210887_m1 | 3.36716228 | 0.00708227 |
| IRF7-Hs01014809_g1 | 4.436197733 | 0.0026576 |
| PRKCD-Hs01090047_m1 | 6.150146209 | 0.00715033 |
| EGR1-Hs00152928_m1 | 12.14994341 | 0.00964639 |
| CDKN2D-Hs00176481_m1 | 12.45349613 | 0.000485024 |
| TERF2-Hs00194619_m1 | 12.80271736 | 0.000881484 |
| ERBB2-Hs01001580_m1 | 15.95378284 | 0.0181667 |
| BCL2L2-Hs00187848_m1 | 18.08373396 | 0.000108308 |
| CDC25c-Hs00156411_m1 | 18.90445806 | 0.000364651 |
| TBX3-Hs00195612_m1 | 21.09668512 | 0.0258012 |
| ETS2-Hs00232009_m1 | 24.25076654 | 0.000500061 |
| BCL2-Hs04986394_s1 | 33.92877452 | 0.0134414 |
| MAP2K6-Hs00992389_m1 | 43.21951674 | 0.000995242 |
| ALDH1A3-Hs00167476_m1 | 94.31430666 | 0.00528085 |
| TBX2-Hs00911929_m1 | 99.05195302 | 0.000229732 |
| SERPINB2-Hs01010736_m1 | 1191.167249 | 0.000307826 |
| IRF5-Hs00158114_m1 | 1191.772334 | 0.000334141 |

**Supplementary File 3c.** Non-DE genes in RG-7112.

| **Decrease** |
| --- |
| **Increase** |

| **Target Name** | **Fold change Average in RG-7112 / CTRL** | **p value** |
| --- | --- | --- |
| FN1-Hs00365052_m1 | 0.004340285 | 0.488806 |
| SOD2-Hs00167309_m1 | 0.011321093 | 0.899049 |
| VIM-Hs00185584_m1 | 0.017053838 | 0.647675 |
| IGFBP3-Hs00365742_g1 | 0.019884602 | 0.665976 |
| CALR-Hs00189032_m1 | 0.025096473 | 0.10989 |
| CD44-Hs01075861_m1 | 0.026781892 | 0.987157 |
| IGFBP7-Hs00266026_m1 | 0.042077737 | 0.947936 |
| COL3A1-Hs00943809_m1 | 0.046606807 | 0.652019 |
| HIF1A-Hs00153153_m1 | 0.053306105 | 0.747794 |
| SPARC-Hs00234160_m1 | 0.055386997 | 0.968579 |
| HSP90AA1-Hs00743767_sH | 0.056066232 | 0.903797 |
| THBS1-Hs00962908_m1 | 0.074553781 | 0.432624 |
| CEBPB-Hs00942496_s1 | 0.137323143 | 0.504241 |
| IL6-Hs00174131_m1 | 0.155298828 | 0.903973 |
| SERPINE1-Hs01126606_m1 | 0.163176802 | 0.848188 |
| COL1A1-Hs00164004_m1 | 0.16384729 | 0.468753 |
| SOD1-Hs00533490_m1 | 0.185270188 | 0.96702 |
| PCNA-Hs00427214_g1 | 0.229258072 | 0.0680976 |
| CCND1-Hs00765553_m1 | 0.322843922 | 0.65941 |
| PLAU-Hs01547054_m1 | 0.331035543 | 0.682536 |
| TGFB1-Hs00998133_m1 | 0.345435461 | 0.355062 |
| CITED2-Hs01897804_s1 | 0.350727303 | 0.868421 |
| ID1-Hs03676575_s1 | 0.395965624 | 0.198869 |
| BCL2L1-Hs00236329_m1 | 0.461364432 | 0.0582994 |
| CREG1-Hs00355412_m1 | 0.514174754 | 0.982492 |
| CDK4-Hs01565683_g1 | 0.618176903 | 0.403128 |
| ETS1-Hs00428293_m1 | 0.735078211 | 0.570344 |
| CDK6-Hs01026371_m1 | 0.775422083 | 0.610591 |
| AKT1-Hs00178289_m1 | 0.870969493 | 0.178615 |
| MAP2K3-Hs00177127_m1 | 0.909691564 | 0.384511 |
| GSK3B-Hs01047719_m1 | 1.028182825 | 0.808768 |
| HRAS-Hs00978050_g1 | 1.067710503 | 0.406721 |
| NFKB1-Hs00765730_m1 | 1.174572171 | 0.450999 |
| IRF3-Hs01547283_m1 | 1.294617412 | 0.325561 |
| MORC3-Hs00899726_m1 | 1.33084989 | 0.705915 |
| ABL1-Hs01104728_m1 | 1.467197154 | 0.154936 |
| TGFB1I1-Hs00210887_m1 | 1.602231007 | 0.599583 |
| MYC-Hs00153408_m1 | 1.60971692 | 0.240054 |
| IGF1R-Hs00609566_m1 | 1.774160593 | 0.532204 |
| GLB1-Hs01035168_m1 | 1.991231412 | 0.312897 |
| BMI1-Hs00995536_m1 | 2.043490199 | 0.321228 |
| TP53-Hs01034249_m1 | 2.180464296 | 0.245246 |
| MAP2K1-Hs00983247_g1 | 2.307518626 | 0.580503 |
| CHEK1-Hs00967506_m1 | 2.418303032 | 0.80099 |
| IRF7-Hs01014809_g1 | 2.439504029 | 0.446787 |
| CDKN2B-Hs00793225_m1 | 2.979658834 | 0.989828 |
| PRKCD-Hs01090047_m1 | 3.125494103 | 0.344976 |
| CDKN2A-Hs00923894_m1 | 3.198562277 | 0.97347 |
| PTEN-Hs02621230_s1 | 3.221589351 | 0.370926 |
| ING1-Hs01941057_u1 | 3.253762425 | 0.96518 |
| RB1-Hs01078066_m1 | 3.69005956 | 0.710407 |
| CCNA2-Hs00996788_m1 | 4.263254412 | 0.140668 |
| E2F3-Hs00605457_m1 | 4.279928943 | 0.194735 |
| SIRT1-Hs01009005_m1 | 4.364398759 | 0.174875 |
| PIK3CA-Hs00907957_m1 | 4.460722833 | 0.811473 |
| TWIST1-Hs01675818_s1 | 4.705575359 | 0.284638 |
| CDK2-Hs01548894_m1 | 4.753796557 | 0.0587869 |
| NBN-Hs01039836_m1 | 5.019831212 | 0.612132 |
| RBL2-Hs00180562_m1 | 5.136623927 | 0.489186 |
| EGR1-Hs00152928_m1 | 5.155479391 | 0.322446 |
| TP53BP1-Hs00996818_m1 | 5.527193313 | 0.453862 |
| ATM-Hs01112355_g1 | 6.155009264 | 0.422256 |
| BCL2L2-Hs00187848_m1 | 7.419899375 | 0.522276 |
| TBX3-Hs00195612_m1 | 8.000003074 | 0.472948 |
| TERF2-Hs00194619_m1 | 8.316692748 | 0.285105 |
| CDKN1B-Hs01597588_m1 | 8.571104306 | 0.748943 |
| ERBB2-Hs01001580_m1 | 8.60760948 | 0.23299 |
| CCNE1-Hs01026536_m1 | 10.95003853 | 0.0955195 |
| ETS2-Hs00232009_m1 | 13.14611642 | 0.123734 |
| CDKN2C-Hs00176227_m1 | 14.75985124 | 0.0807383 |
| TBX2-Hs00911929_m1 | 21.50841931 | 0.580586 |
| SERPINB2-Hs01010736_m1 | 22.36030171 | 0.936443 |
| IGFBP5-Hs00181213_m1 | 27.52882886 | 0.233171 |
| BCL2-Hs04986394_s1 | 29.00663916 | 0.333629 |
| ALDH1A3-Hs00167476_m1 | 47.67868278 | 0.579648 |
| IGF1-Hs01547656_m1 | 57.67025026 | 0.672141 |
| CHEK2-Hs00200485_m1 | 72.5949038 | 0.492235 |
| CDKN1C-Hs00175938_m1 | 182.0577012 | 0.829068 |
| ROS1-Hs00177228_m1 | 188.2693706 | 0.890908 |
| IRF5-Hs00158114_m1 | 197.1434008 | 0.558927 |
| MAP2K6-Hs00992389_m1 | 344.6200894 | 0.487212 |
| NOX4-Hs00418356_m1 | 495.1797467 | 0.815625 |
| NTRK3-Hs00983871_m1 | 1947.329308 | 0.699694 |

**Supplementary File 3d.** Non-DE genes in o-Vanillin.

| **Decrease** |
| --- |
| **Increase** |

| **Target Name** | **Fold change Average in O-Vanillin / CTRL** | **p value** |
| --- | --- | --- |
| COL3A1-Hs00943809_m1 | 0.024124278 | 0.119285 |
| IGFBP3-Hs00365742_g1 | 0.024511705 | 0.253964 |
| CALR-Hs00189032_m1 | 0.030532664 | 0.595177 |
| IGFBP7-Hs00266026_m1 | 0.033306427 | 0.72376 |
| SPARC-Hs00234160_m1 | 0.049001389 | 0.851548 |
| HSP90AA1-Hs00743767_sH | 0.050923105 | 0.745989 |
| HIF1A-Hs00153153_m1 | 0.068570014 | 0.219955 |
| SOD1-Hs00533490_m1 | 0.159658446 | 0.972407 |
| CITED2-Hs01897804_s1 | 0.165002798 | 0.216478 |
| IL6-Hs00174131_m1 | 0.171439093 | 0.710533 |
| COL1A1-Hs00164004_m1 | 0.186048311 | 0.255593 |
| ID1-Hs03676575_s1 | 0.199541441 | 0.937302 |
| PCNA-Hs00427214_g1 | 0.223385318 | 0.107749 |
| CEBPB-Hs00942496_s1 | 0.273200925 | 0.388586 |
| CDK4-Hs01565683_g1 | 0.434052566 | 0.179506 |
| SERPINE1-Hs01126606_m1 | 0.443498833 | 0.0644238 |
| CREG1-Hs00355412_m1 | 0.506755467 | 0.890009 |
| CDKN1A-Hs00355782_m1 | 0.783578481 | 0.313523 |
| MDM2-Hs01066930_m1 | 0.815917354 | 0.496983 |
| GSK3B-Hs01047719_m1 | 1.076994611 | 0.905942 |
| IGF1R-Hs00609566_m1 | 1.555456887 | 0.876913 |
| BMI1-Hs00995536_m1 | 1.676721751 | 0.798902 |
| MORC3-Hs00899726_m1 | 1.732602373 | 0.334395 |
| MAP2K1-Hs00983247_g1 | 2.08429382 | 0.855847 |
| GLB1-Hs01035168_m1 | 2.172948736 | 0.142358 |
| TP53-Hs01034249_m1 | 2.299786488 | 0.16202 |
| MYC-Hs00153408_m1 | 2.509016777 | 0.0621116 |
| RB1-Hs01078066_m1 | 2.702357844 | 0.394831 |
| SIRT1-Hs01009005_m1 | 2.769287597 | 0.273404 |
| PTEN-Hs02621230_s1 | 2.798748657 | 0.683617 |
| TWIST1-Hs01675818_s1 | 3.049087597 | 0.767845 |
| NBN-Hs01039836_m1 | 3.292091508 | 0.296064 |
| PIK3CA-Hs00907957_m1 | 3.302093615 | 0.55577 |
| ING1-Hs01941057_u1 | 3.669249974 | 0.506152 |
| TP53BP1-Hs00996818_m1 | 3.952600883 | 0.216849 |
| E2F3-Hs00605457_m1 | 4.285500089 | 0.17144 |
| CDK2-Hs01548894_m1 | 4.834294816 | 0.280465 |
| CDKN1B-Hs01597588_m1 | 5.481382204 | 0.0816086 |
| RBL2-Hs00180562_m1 | 5.533616087 | 0.220979 |
| ATM-Hs01112355_g1 | 5.636585939 | 0.309837 |
| RBL1-Hs00765700_m1 | 6.054935484 | 0.068613 |
| CDKN2B-Hs00793225_m1 | 7.311803675 | 0.0607971 |
| CCNE1-Hs01026536_m1 | 8.162041521 | 0.408224 |
| E2F1-Hs00153451_m1 | 10.91296887 | 0.150587 |
| IGF1-Hs01547656_m1 | 26.82235498 | 0.257233 |
| CHEK2-Hs00200485_m1 | 32.80216414 | 0.098609 |
| IGFBP5-Hs00181213_m1 | 33.45295298 | 0.206856 |
| CDKN1C-Hs00175938_m1 | 134.5927466 | 0.541087 |
| ROS1-Hs00177228_m1 | 394.3410328 | 0.450937 |
| NOX4-Hs00418356_m1 | 427.8713173 | 0.484347 |
| NTRK3-Hs00983871_m1 | 4006.421901 | 0.370474 |
