# Supplementary material for: Senotherapeutic drugs for human intervertebral disc degeneration and low back pain
Source: eLife. 2020 Aug 21;9:e54693. doi: 10.7554/eLife.54693 (PMC7442487; doi:10.7554/eLife.54693)
Supplement: Supplementary file 4. — (a) O-Vanillin network scores, molecules and top diseases. Physical function analysis using IPA of o-Vanillin treated NP pellets generated three networks which are ordered by a score denoting significance. The highest-scoring network, which comprises 37 proteins in our list, revealed significant changes in cell death and survival, neurological disease, organismal Injury and abnormalities. In addition, the other networks revealed changes in cancer, cellular movement, connective tissue development and function, cell cycle, organismal injury and abnormalities. (b) RG-7112 network scores, molecules and top diseases. Physical function analysis using IPA of RG-7112 treated NP pellets generated a single network which is ordered by a score denoting significance of 21. This network reveals significant changes in cell cycle, cell death and survival, connective tissue development and function. [file elife-54693-supp4.docx]

**Supplementary File 4a.** **O-Vanillin networks scores, molecules and top diseases.** Physical function analysis using IPA of o-Vanillin treated NP pellets generated three networks which are ordered by a score denoting significance. The highest-scoring network, which comprises 37 proteins in our list, revealed significant changes in cell death and survival, neurological disease, organismal Injury and abnormalities. In addition, the other networks revealed changes in cancer, cellular movement, connective tissue development and function, cell cycle, organismal injury and abnormalities.

| **Network ID** | **Molecules in Network** | **Score** | **Focus Molecules** | **Top Diseases and Functions** |
| --- | --- | --- | --- | --- |
| 1 | 14-3-3,ABL1,BCL2L1, BCL2L2, BCR(complex),  caspase,Caspase3/7,Cbp/p300,CCNB1,CD3,CDC25C,CDK6,CDKN2A,CDKN2C,CDKN2D,CHEK1,CyclinA,E2f,ETS1,ETS2,Gsk3,Jnk,MAP2K3,MAP2K6,MAPK14,NFkB(complex),PARP,PI3K(complex),PP2A,PRKCD,Rock,SERPINB2,Smad,SOD2,STAT5a/b | 37 | 18 | [Cell Death and Survival, Neurological Disease, Organismal Injury and Abnormalities] |
| 2 | Akt,Ap1,calpain,CD44,CG,  Creb,EGR1,ERK,ERK1/2,FN, Focal-adhesion-kinase,HRAS, Hsp27,Igm,IL12(complex),IRF3,IRF5,JAK,LDL,MAP2K1/2,Mapk,Mek,P38MAPK,PDGFB,PI3K(family),Pkc(s),PLAU,SRC(family),TBX2,TBX3,TGFB1,TGFB1I1,THBS1,VEGF,  VIM | 24 | 13 | [Cancer, Cellular Movement, Connective Tissue Development and Function] |
| 3 | 26sProteasome,ABCD3, AKT1,BCL2,CBR3AS1, CCNA2,CCND1,CDC37L1,ERBB2,estrogen-receptor, Hdac, Histoneh3,Histoneh4,Hsp70, Hsp90,IFNBeta,IgG, Interferon alpha,IRF7,LAP3, LGALS7/ LGALS7B,MUS81, NDUFA7, NDUFB1,NDUFB7,NFKB1,PI3K,p85,PPL,RNApolymeraseII,RPL3,RPS2,TCF,TCR,TERF2,TRIM32 | 13 | 8 | [Cancer, Cell Cycle, Organismal Injury and Abnormalities] |

**Supplementary File 4b. RG-7112 networks scores, molecules and top diseases.** Physical function analysis using IPA of RG-7112 treated NP pellets generated a single network which is ordered by a score denoting significance of 21. This network reveals significant changes in cell cycle, cell death and survival, connective tissue development and function.

| **Network ID** | **Molecules in Network** | **Score** | **Focus Molecules** | **Top Diseases and Functions** |
| --- | --- | --- | --- | --- |
| 1 | Akt,ALDH3A1,CCNB1,CDC25C,CDKN1A,CDKN2D,CyclinA,CyclinE,E2F1,FOXG1, FRMD6,GNL3L, GSTA4,Histoneh3,Histone h4,JMY,MAGEA11, MAPK14,MDM2,MSI1, NFkB(complex),P38 MAPK,PHF8,PI3K (complex),PYHIN1,RASSF3,Rb,RBL1,RBM38,RNF126,SP1,STX8,SYF2, USP42,ZBTB5 | 21 | 8 | [Cell Cycle, Cell Death and Survival, Connective Tissue Development and Function] |
